# Case Report: Differential diagnosis and clinical management of isolated prolonged activated partial thromboplastin time

**DOI:** 10.3389/fimmu.2026.1735963

**Published:** 2026-03-06

**Authors:** Lu Liu, Dongmei Guo

**Affiliations:** Department of Hematology, Qilu Hospital (Qingdao) of Shandong University, Qingdao, China

**Keywords:** acquired hemophilia, antiphospholipid antibodies, APTT, case report, lupus anticoagulant

## Abstract

An unexpected, isolated prolongation of activated partial thromboplastin time (APTT) frequently poses diagnostic challenges in coagulation laboratories. This article presents two patients exhibiting isolated prolonged APTT, one with significant bleeding manifestations and another without bleeding symptoms. The first patient was diagnosed with acquired hemophilia A (AHA), whereas antiphospholipid antibodies (aPLs) were suspected to be responsible for the isolated prolonged APTT in the second patient. Accurate identification of the underlying cause of isolated prolonged APTT is crucial for proper diagnosis and subsequent therapeutic management. Furthermore, we summarize potential causes of isolated prolonged APTT and propose a simplified diagnostic algorithm to distinguish between lupus anticoagulants (LA) (more common) and coagulation factor deficiencies (less common). This report aims to provide insights into the clinical management of similar cases in the future.

## 1 Introduction

Prolongation of activated partial thromboplastin time (APTT) is frequently observed clinically, generally indicating deficiencies or inhibitors in the intrinsic coagulation pathway. An extended APTT serves as a critical indicator of coagulation disorders and is essential for diagnosing various hematological diseases (1). Recent studies identified multiple causes for prolonged APTT, including intrinsic pathway factors deficiencies, anticoagulant medications, autoimmune antibodies, and drug-induced coagulation abnormalities. These diverse etiologies require distinct treatment strategies, complicating diagnosis. APTT prolongation caused by lupus anticoagulant (LA) is typically associated with an increased risk of future thrombosis rather than bleeding (2, 3). Misinterpretation of isolated prolonged APTT may result in unnecessary or inappropriate therapeutic interventions. Here, we report two cases of isolated prolonged APTT encountered in our hospital and review pertinent literature to enhance diagnostic accuracy and clinical decision-making.

## 2 Case 1

A 68-year-old male patient was admitted to our hospital in November 2023 due to generalized ecchymosis. He was diagnosed with atopic dermatitis in 2022 and treated with hydrocortisone acetate ointment. The patient underwent surgery for hemorrhoids in 2010 and nasal septum surgery in 2021, without abnormal bleeding during these procedures. Previous coagulation tests were normal. He denied additional medication use, relevant medical conditions, or history of blood transfusions. There was no family history of genetic disorders or malignancies. Laboratory testing revealed a white blood cell (WBC) count of 10.93 × 10^9^/L, hemoglobin level of 75 g/L, and platelet count of 265 × 10^9^/L. The patient’s prothrombin time (PT), thrombin time (TT), and fibrinogen levels were within normal limits. APTT was prolonged at 97.1 seconds (reference range: 28–43.5 seconds). The APTT ratio was 2.71 (reference range: 0.8–1.2).All reagents and equipment used for coagulation tests were obtained from Diagnostica Stago (France), and coagulation analysis was performed using a STA-R Max automatic analyzer. Computed tomography (CT) imaging revealed a hematoma in the right iliopsoas muscle. Biochemical screening, thyroid function tests, autoimmune antibody panels, tumor markers, and vascular ultrasound results were normal. LA was detected with the modified dilute Russell viper venom time (dRVVT) method (4) using a STA-R Max automatic analyzer. LA testing was negative. The mixing test was carried out as previously described in detail (5, 6). Assessment of APTT correction was performed using the index of circulating anticoagulant (ICA) method, also known as the Rosner index (RI) (7, 8). If the difference exceeds 3 seconds in time incubation mixing study, this suggests the presence of time- and temperature-dependent inhibitors (9). Mixing studies showed ICA below 15% after immediate mixing in this patient. Time-incubation mixing studies indicated that prolonged APTT resulted from coagulation factor inhibitors (**Table 1**). Chromogenic assay showed factor VIII activity below 1.0% (reference range: 50%–150%), while other coagulation factors were normal. Factor VIII inhibitor screening (Bethesda method) revealed an inhibitor level of 36.8 BU/mL. All reagents and equipment used to measure factor activities and inhibitors were obtained from Sysmex Corporation(Japan). The patient was diagnosed with acquired hemophilia A (AHA) complicated by hemorrhagic anemia. Treatment commenced with methylprednisolone (0.8 mg/kg, dose tapered after stabilization), cyclophosphamide (1 g, single dose), and rituximab (100 mg weekly for 4 weeks). After 4 weeks of treatment, the patient’s hemoglobin concentration and APTT normalized. Factor VIII activity increased to 172.1%, and factor VIII inhibitor was negative after 2 months.

**Table 1:** Results of coagulation parameters.

Indicators (Unit)/Test	PT, s	APTT, s	TT, s	FIB, g/L	APTT immediate mixing study, %	APTT time incubating mixing study
Case 1	13.5	97.1	15.2	3.0	29.12%	23.5s extension
Case 2	13.8	64.6	16.5	3.31	25.54%	3.3s extension
Reference value/Judgment criteria	11.0-14.5	28.0-43.5	14.0-21.0	2.0-4.0	ICA >15%: not corrected; ICA <10%: corrected	<3s extension: LA; >3s extension: factor inhibitors

APTT, activated partial thromboplastin time; FIB, fibrinogen; ICA, index of circulating anticoagulant; LA, lupus anticoagulant; PT, prothrombin time; TT, thrombin time.

## 3 Case 2

A 50-year-old female patient was admitted to our hospital in November 2024 due to recurrent gum bleeding, which resolved spontaneously. No other bleeding symptoms were reported. She denied additional medical conditions, previous surgeries, or blood transfusions. There was no known genetic or family history of malignancy. Routine laboratory examinations upon admission, including hematology, biochemical screening, thyroid function tests, tumor markers, and vascular ultrasound, showed normal results. APTT was prolonged at 64.6 seconds (reference range: 28–43.5 seconds). The APTT ratio was 1.8 (reference range: 0.8–1.2).The LA screening ratio was 1.42 (reference range: 0–1.2), the LA confirmation ratio was 1.11, and their normalized ratio was 1.28 (reference range: 0–1.2). Anticardiolipin (aCL) antibodies and anti-β2-glycoprotein I (β2-GPI) antibody were detected using chemiluminescence immunoassay (Centuryyis, China). aCL antibody IgM levels were mildly elevated at 10.46 MPLU/mL (reference range: 1–10 MPLU/mL), while aCL antibody IgG (1.01 GPLU/mL) and IgA (0.75 APLU/mL) were negative. Anti-β2-GPI antibody IgM (4.96 RU/mL), IgG (<2.0 RU/mL), and IgA (<2.0 RU/mL) were negative. All other serological tests for autoimmune antibodies, including anti-Jo-1, anti-mitochondrial, anti-glomerular basement membrane, anti-proteinase 3, anti-myeloperoxidase, anti-double-stranded DNA, anti-nucleosome, and antinuclear antibodies, were negative. These antibodies were detected with enzyme linked immunosorbent assay (ELISA) method (AESKU, Germany). Coagulation factor activities, as well as von Willebrand factor (vWF) antigen and activity, were within normal limits. The reagents and equipment used were identical to those described in Case 1. The patient did not fulfill the diagnostic criteria for any autoimmune disease. We considered antiphospholipid antibodies (aPLs) to be the likely cause of isolated prolonged APTT in this patient. Given the absence of coagulation factor abnormalities and significant bleeding manifestations, the patient remains under observation without specific treatment. As the patient did not return for follow-up, the subsequent results of APTT, LA, aCL and anti−β2-GPI antibodies didn’t be obtain.

## 4 Discussion

APTT is a widely utilized coagulation assay, and isolated prolongation of APTT with normal PT is relatively common in clinical practice. Both acquired factor VIII (FVIII) inhibitors and aPLs can prolong APTT, though they represent distinctly different clinical scenarios. Distinguishing between these two entities can be challenging for both clinicians and laboratory personnel, regardless of whether the clinical manifestation is bleeding or thrombosis.

AHA is a rare disorder characterized by circulating autoantibodies against FVIII, with bleeding as the primary clinical manifestation. It typically presents as spontaneous bleeding or abnormal bleeding during surgery or trauma, in individuals without prior bleeding disorders or family history (10). AHA can occur at any age but predominantly affects elderly individuals aged 64–78 years. Some cases are associated with autoimmune diseases (16.6%), malignancies (44%), or pregnancy (4%), whereas others are idiopathic with no identifiable cause (11–13). Clinical presentations of bleeding in AHA are highly heterogeneous (14). Mild cases may remain asymptomatic, identified incidentally due to prolonged APTT on routine tests. Severe cases can involve acute, life-threatening bleeding, including extensive subcutaneous hematomas or spontaneous hemorrhages. Unlike congenital hemophilia, joint bleeding is uncommon in AHA; bleeding primarily involves the skin, mucous membranes, subcutaneous tissues, intermuscular spaces, visceral organs, and occasionally intracranial regions (15). In AHA, bleeding severity does not correlate directly with antibody titers, and mortality rates range between 9% and 22% (16). Deaths usually occur within one week of onset, primarily due to gastrointestinal or pulmonary hemorrhages. AHA is often underdiagnosed due to its rarity and limited clinical awareness, particularly when patients initially present to surgical units, resulting in delayed diagnosis and poorer outcomes. In the first case described here, the patient was an elderly male with a history of atopic dermatitis, presenting with an acute muscle hematoma and significantly prolonged APTT, prompting clinicians to strongly suspect AHA during diagnostic evaluation.

LA, aCL antibodies, and anti-β2-GPI antibodies are collectively termed aPLs and are widely used as laboratory markers for antiphospholipid syndrome (APS) (17). LA shows the highest detection rate and has a stronger association with thrombotic events than aCL or anti−β2−GPI antibodies (18). Although LA exhibits anticoagulant effects *in vitro*, manifested as prolonged APTT, it exerts procoagulant effects *in vivo*, thereby increasing thrombotic risk (19). aPLs are autoantibodies directed against phospholipids or phospholipid−binding proteins (20) and represent important risk factors for thrombosis and adverse pregnancy outcomes. aPLs have been detected in malignancies, infections, drug−related conditions, and even in healthy individuals (21, 22).Mustonen et al. (23) conducted a prospective study involving 119 asymptomatic patients with persistently positive aPLs, of whom 61% had autoimmune diseases such as systemic lupus erythematosus (SLE). During a median follow-up period of 7.5 years, the incidence of thrombotic events was observed. Results indicated that all patients experiencing thrombosis had a history of SLE. Patients with SLE and aPLs demonstrated a higher incidence of thrombosis compared to patients positive for aPLs alone. Therefore, clinicians should consider the possibility of autoimmune disease in patients presenting with thrombotic events associated with aPLs. Case 2 in our report involved a middle-aged female patient without venous thrombotic events or autoimmune disease, presenting with prolonged and uncorrectable APTT and positive LA and aCL antibodies, providing diagnostic clues for the condition.

**Figure 1:**
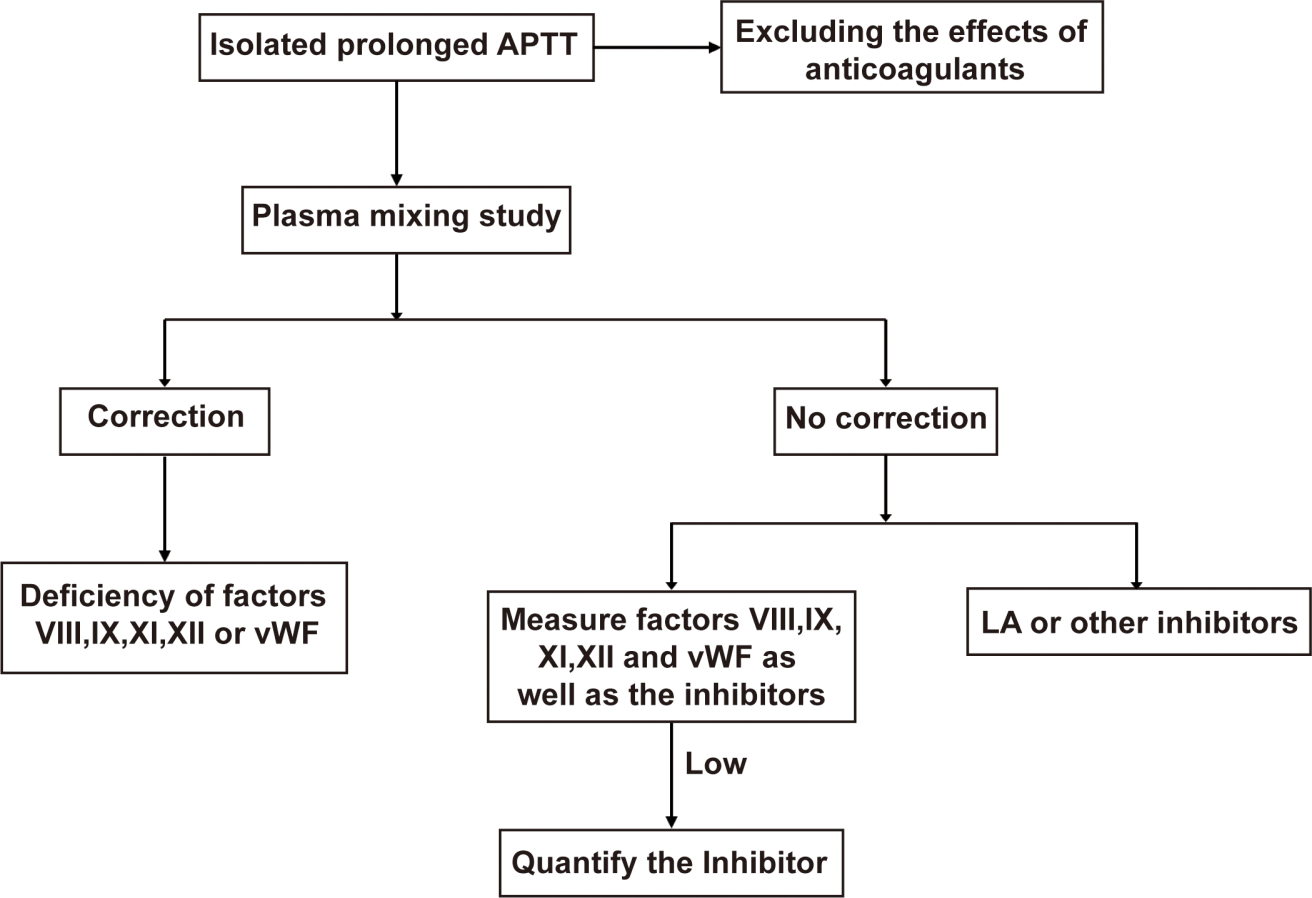
Diagnostic flowchart of isolated prolonged APTT. If the plasma mixing study is correction, measure factors VIII, IX, XI, XII and vWF. No correction of plasma mixing study indicates the presence of inhibitors. APTT, activated partial thromboplastin time; vWF, von Willebrand factor; LA, lupus anticoagulant.

When patients present with isolated prolonged APTT without clinical symptoms, distinguishing between AHA and aPLs depends on laboratory testing. After excluding heparin interference, a plasma mixing study is performed by mixing the patient’s plasma with normal plasma (1:1 ratio) and incubating at 37 °C for 2 hours, followed by repeated APTT measurement (24). If the prolonged APTT does not correct, activities of coagulation factors FVIII, FIX, FXI, and FXII should be evaluated. In AHA, FVIII activity is significantly reduced, often severely or completely. One-stage clot-based assays may be affected by LA, whose interference can mimic FVIII deficiency (25, 26). However, LA does not interfere with chromogenic FVIII activity assays. In cases of high-titer aPLs, FVIII activity measured by the one-stage assay may appear decrease, but normal results are obtained with chromogenic assays (27). Occasionally, patients may simultaneously have FVIII inhibitors and LA. In complex situations, FVIII antibody detection by ELISA may help differentiate FVIII inhibitors from LA (28, 29). The diagnostic algorithm for isolated prolonged APTT is illustrated in **Figure 1**.

In summary, we reported two cases of isolated prolonged APTT arising from different etiologies. Not all instances of prolonged APTT result in increased bleeding risk. Any patient exhibiting isolated prolonged APTT should undergo further assessment to distinguish anticoagulants, coagulation factor deficiencies, or aPLs. This report aims to provide diagnostic guidance and assist clinicians in making appropriate clinical and laboratory decisions.

## 5 Patient perspective

The patient in Case 1 initially presented with ecchymosis and a hematoma in the right iliopsoas muscle. LA was negative, FVIII activity was significantly reduced, and FVIII inhibitor testing was positive. The patient was diagnosed with AHA and treated with methylprednisolone, cyclophosphamide, and rituximab, after which hemoglobin concentration, APTT, and FVIII activity returned to normal.

In Case 2, aCL IgM antibodies and LA were positive, whereas coagulation factor activities remained within normal limits. The patient did not meet the diagnostic criteria for any autoimmune disease and presented without abnormal bleeding symptoms. She remains under clinical follow-up without specific treatment.

## Data Availability

The raw data supporting the conclusions of this article will be made available by the authors, without undue reservation.
